# Effects of Preoperative Telerehabilitation on Muscle Strength, Range of Motion, and Functional Outcomes in Candidates for Total Knee Arthroplasty: A Single-Blind Randomized Controlled Trial

**DOI:** 10.3390/ijerph18116071

**Published:** 2021-06-04

**Authors:** Jungae An, Ho-Kwang Ryu, Suk-Joo Lyu, Hyuk-Jong Yi, Byoung-Hee Lee

**Affiliations:** 1 Graduate School of Physical Therapy, Sahmyook University, Seoul 01795, Korea; jungaean@hotmail.com; 2 Department of Orthopedic Surgery, Seoul Now Hospital, Seongnam 13591, Korea; gratie@daum.net (H.-K.R.); valencia1@naver.com (S.-J.L.); 3 Department of Physical Therapy, Seoul Now Hospital, Seongnam 13591, Korea; bjong2@hanmail.net; 4 Department of Physical Therapy, Sahmyook University, Seoul 01795, Korea

**Keywords:** osteoarthritis, prehabilitation, telerehabilitation, total knee arthroplasty

## Abstract

This study aims to investigate the effect of a preoperative telerehabilitation (PT) program on muscle strength, knee range of motion (ROM), and functional outcomes in candidates for total knee arthroplasty (TKA). Sixty patients (all women, mean age 70.53 ± 2.7 years) scheduled for bilateral TKA participated in this study. The PT and preoperative patient education (PE) groups participated in a 3-week intensive exercise program (30 min/session, 2 times/day, 5 days/week), whereas the control group received the usual care before TKA. Quadriceps muscle strength, Western Ontario and McMaster Universities Osteoarthritis Index (WOMAC), ROM of knee flexion, pain, and time up-and-go (TUG) test time were evaluated at 4 weeks preoperatively, post-interventionally, and 6 weeks after TKA. Significant differences were found in the time-by-group interaction for 60°/s extension peak torque [F(4, 100) = 2.499, *p* = 0.047, *η^2^_p_* = 0.91], 180°/s extension peak torque [F(4, 100) = 3.583, *p* = 0.009, *η^2^_p_* = 0.125], ROM [F(4, 100) = 4.689, *p* = 0.002, *η^2^_p_* = 0.158], TUG time [F(4, 100) = 7.252, *p* < 0.001, *η^2^_p_* = 0.225], WOMAC pain [F(4, 100) = 9.113, *p* < 0.001, *η^2^_p_* = 0.267], WOMAC functional outcome [F(4, 100) = 6.579, *p* < 0.001, *η^2^_p_* = 0.208], and WOMAC total score [F(4, 100) = 10.410, *p* < 0.001, *η^2^_p_* = 0.294]. The results of this study demonstrate the early benefits of a PT program in elderly female patients with end-stage osteoarthritis. The PT program improved muscle strength, ROM, and functional outcomes before TKA, which contributed to better functional recovery after TKA.

## 1. Introduction

Osteoarthritis is a common chronic disease in elderly people aged >65 years, and patients with knee osteoarthritis experience pain, swelling, limited joint mobility, and stiffness due to inflammation of the synovial joint [1,2,3]. To improve the pain and physical function of patients with knee osteoarthritis, previous studies have proposed effective physical therapy interventions, such as manual therapy, physical agent treatment, use of brace and orthoses, aerobic walking, strengthening training, balance training, home-based exercises, self-management programs, and weight reduction programs [4]. However, although these physical therapy interventions have demonstrated valuable results, patients with end-stage knee osteoarthritis still consider undergoing total knee arthroplasty (TKA) to improve their physical performance and quality of life [5].

TKA is a surgical procedure that replaces a damaged knee with an artificial prosthesis. It has been suggested as an effective solution for decreasing pain and for the recovery of physical function in patients with end-stage knee osteoarthritis, owing to advances in artificial prosthesis design and surgical technology over the past decades [6,7]. By 2030, the number of primary TKA procedures performed is expected to increase by 673% to 3.48 million cases in the United States [8]. Meanwhile, primary TKA is performed in >75,000 cases annually in Korea (Statistics Korea). Despite reports about relevant outcomes in terms of pain, functional recovery, and quality of life after a successful TKA, patients still complain of persistent impairments in physical function, muscle strength, and balance ability until the first year after surgery [9,10]. Prior studies have demonstrated a wide range of physical therapy interventions that aim to achieve efficient physical and functional outcomes after TKA [11]. Preoperative muscle strength and physical function may contribute to postoperative outcomes; however, most physical therapy interventions for TKA rehabilitation are focused on the postoperative period [12,13].

The concept of prehabilitation (or preoperative rehabilitation), including medical or behavioral support interventions such as exercise, physical therapy, and lifestyle modifications performed before surgery, has been implemented in patients with cancer, cardiopulmonary diseases, and musculoskeletal diseases, with reported beneficial effects [14]. Recent studies have found that a preoperative rehabilitation program for patients scheduled to undergo TKA improves postoperative pain, range of motion (ROM), balance ability, stiffness, muscle strength, length of hospital stay (LOS), and health-related quality of life; however, the effectiveness of preoperative interventions varied with intervention protocols such as intensity, frequency, the content of the program, and duration of intervention, and is still controversial [15,16,17,18,19].

The implementation of telerehabilitation in physical therapy can enable the remote delivery of personalized assessment and treatment intervention through the use of digital technology, thereby providing the advantages of treatment accessibility and cost reduction for patients living in areas far from rehabilitation facilities [20,21]. The application of telerehabilitation has increased along with technological developments, and previous studies have shown the potential of telerehabilitation as an alternative to hospital-based rehabilitation. In fact, a study on home-based physical therapy through telephone calls demonstrated beneficial effects on pain and physical function in patients with knee osteoarthritis [22]. In addition, telerehabilitation in patients undergoing TKA has been shown to have similar treatment effects to face-to-face treatment in terms of pain relief, ROM of the knee, quadriceps strength, and physical function [23]. Furthermore, a preoperative telerehabilitation program for patients who are candidates for TKA has been shown to provide benefits in terms of early-phase outcomes after TKA; however, the recent study of preoperative-telerehabilitation has reported no effect on muscle strength and functional outcomes and the efficacy of tele-prehabilitation is lacking evidence from multiple perspectives [24,25]. Moreover, there were no preoperative telerehabilitation programs that combined intensive training for candidates who are waiting only for a short period for TKA in various medical systems. The purpose of this study is to investigate the effects of a preoperative telerehabilitation program with intensive training for 3 weeks on muscle strength, ROM, and functional outcomes in patients undergoing TKA.

## 2. Materials and Methods

### 2.1. Participants

Patients who were scheduled to undergo primary TKA for the treatment of advanced knee osteoarthritis were recruited from an orthopedic surgery rehabilitation hospital. The inclusion criteria for participation in this preoperative telerehabilitation program were as follows: (1) scheduled bilateral TKA, (2) age ranging from 65 to 75 years, (3) efficient control of comorbid diseases, (4) ability to walk over 10 m without help, (5) average vision and hearing, (6) consent to voluntary participation in the preoperative rehabilitation program with prior consent from the surgeon, (7) without cognitive impairment and with good communication ability, and (8) familiarity with smartphone apps. The exclusion criteria were as follows: (1) medical instability such as uncontrolled hypertension and arrhythmia or an unstable cardiovascular status, (2) inflammatory arthritis, (3) scheduled TKA revision, (4) history of knee surgery within 6 months, (5) neurological disorders (including Parkinson’s disease and stroke), and (6) neurological damage to the lower extremity.

### 2.2. Ethical Statement

Before participation in this study, the test and intervention protocols were fully explained to, and written informed consent was obtained from the patients or their caregivers. This study was conducted in accordance with the Declaration of Helsinki and was approved by the institutional review board of Sahmyook University in the Republic of Korea (2-7001793-AB-N-012019032HR). The protocol of this trial was retrospectively registered with the Clinical Research Information Service of the Republic of Korea (KCT0005800).

### 2.3. Study Design

The present study was a three-arm, parallel-group, single-blind randomized controlled trial. A total of 231 patients who were scheduled to undergo TKA were assessed for eligibility. Of them, 171 patients were excluded. For sample size determination in this study, we performed a power analysis using G*Power (version 3.1.9.4; Heinrich-Heine-Universität, Düsseldorf, Germany, 2019) before participant recruitment. The overall effect size index for all outcome measures and the power of the study were both 0.25; the probability was 0.05; type II error (power 95%) was minimized; the number of groups was 3; and the number of measurements was 3. As the estimated target sample size was 54, we recruited 60 participants who were scheduled for TKA [26]; the random allocation was generated by a research nurse not involved in this study using a computerized random number generator. After the baseline assessment, 60 patients who consented to participate and were eligible for randomization were allocated to the experimental 1 (preoperative telerehabilitation) group, experimental 2 (patient education) group, or control group by each patient choosing one of three envelopes. The group assignments were concealed in envelopes. The assessors and participants were blinded to the group assignments. The assessors who measured all baseline data, post-intervention data, and postoperative outcomes were unaware of the participants’ allocation, and all measurements were performed in an independent room. All interventions were individually performed. The enrollment period was from August 2019 to April 2020.

### 2.4. Procedure

The preoperative telerehabilitation intervention was conducted 3 weeks before the TKA procedure. Therefore, examinations were performed at three time points. Baseline data and post-intervention data were collected at 4 weeks and 1 day before the TKA procedure, respectively. Follow-up was completed at 6 weeks after TKA. These three measurement points were used to evaluate the effect of the preoperative intervention on the patients’ physical function, pain, muscle strength, activities of daily living, and quality of life after TKA.

After the completion of the experimental procedure, all participants underwent TKA with the same type of high-flexion mobile prosthesis (Implantcast; GMBH Lüneburger Schanze, Buxtehude, Germany) with cement using a tricompartmental, minimally invasive quadriceps-sparing technique [27].

**Figure 1:**
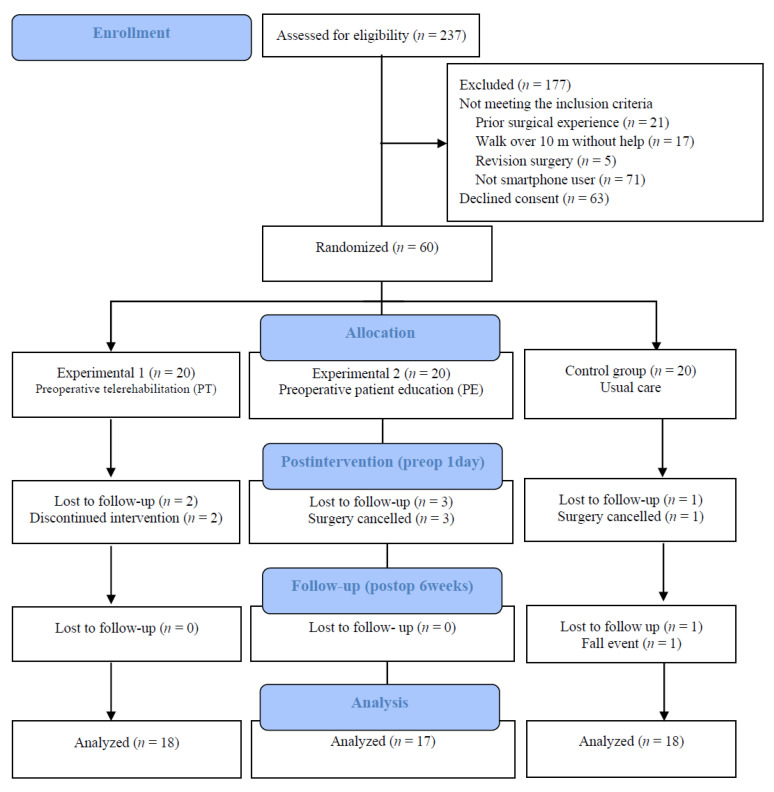
Participant flow diagram.

After TKA, all patients participated in a standardized rehabilitation program for inpatients at an orthopedic rehabilitation hospital for 3 weeks. The postoperative physical therapy program started on the day immediately post-TKA, was performed daily and included continuous passive motion exercises, intermittent pneumatic compression, and cold pack therapy. On the second postoperative day, weight-bearing with the aid of a walker was started after drain removal and wearing a compression stocking, cryotherapy, and manual therapy added for physical therapy. For the first and second weeks, the patients completed knee ROM exercises, ankle pumping, straight leg raises, self-passive knee extension, balance training, and gait training with a walker. In the third week, strength training, endurance exercise, and walking up and down stairs were added (Figure 1).

### 2.5. Interventions

#### 2.5.1. Preoperative Telerehabilitation Group

The intervention group participated in a preoperative telerehabilitation program (30 min/session, 2 times/day, 5 days/week for 3 weeks, for a total of 30 sessions) before TKA. The protocol of the preoperative telerehabilitation program is described in Appendix A. Each session included warm-up, mobility, flexibility, strength, balance, and cool-down exercises. The warm-up and cool-down exercises consisted of knee and ankle ROM exercises and slow walking for 5 min. During the mobility exercises, the patients completed straight leg raises, bridges, leg slides, knee press, passive ROM, and mini squats. After the mobility exercises, the patients performed flexibility exercises consisting of knee extensor stretching, knee flexor stretching, and leg slides. For strengthening exercises that involved knee flexor, knee extensor, and hip abductor training, we used elastic resistance bands such as Thera-Band^®^ (Hygienic Corporation, Akron, OH, USA). The intensity of the resistance was adjusted to a moderate level for each patient. After the strengthening exercises, the patients performed balance exercises that included tandem walk and trunk rotation. These exercises were designed to have medium to low intensity that would not cause pain in the knees of patients with end-stage osteoarthritis. The total exercise time was set to 30 min, and the rest time was set to 20 s. Additional rest time was allowed when fatigue occurred or on a patient’s request. All interventions were performed at home using a smartphone or tablet via a two-way video call. The therapist provided supervision and intervention with real-time visual feedback and verbal cues. The patients participated in a comfortable position using an assistive device that can distribute weight while standing or sitting.

#### 2.5.2. Preoperative Patient Education Group

The preoperative patient education group initially participated in a preoperative education session, which consisted of home exercise safety and the protocol of intervention details for 40 min and thereafter performed a self-home exercise. This non-supervised intervention was performed for 30 min per session, 2 times/day, 5 days/week for 3 weeks, and the exercise timing was adjustable. A physical therapist checked physical condition and provided daily notification, motivation and education of the preoperative exercise via telephone calls once every day. The intervention protocol was the same as the preoperative telerehabilitation program and the patients were encouraged to complete each exercise. The intensity of each exercise was designed to not exacerbate the pain. The exercise details were described in the patient guidebook.

#### 2.5.3. Control Group

The control group received the usual care, such as following the guideline of surgical procedure, postoperative progress monitoring, discharge destination determination, and simple quadriceps exercise intervention. The exercise was recommended to be performed several times daily as the patient’s condition determined.

### 2.6. Primary Outcome Measures

#### Isokinetic Strength Assessment

Quadriceps strength was measured using a dynamometer (Biodex 3 PRO; Biodex Medical Systems Inc., Shirley, NY, USA, 2015). The test–retest reliability showed a high intraclass correlation coefficient (ICC) of 0.947–0.966 in patients who underwent TKA [28]. During the measurement, the patient’s chest, abdomen, thigh, and ankles were fixed with a strap, and the dynamometer rotation axis was aligned with the knee joint axis. Isokinetic force was measured with knee flexion at 60°/s and 180°/s peak torque (N-m) and knee extension at 60/sec° and 180°/s peak torque (N-m) [29]. The average value of all three trials was used for statistical analysis.

### 2.7. Secondary Outcome Measures

#### 2.7.1. Western Ontario and McMaster Universities Osteoarthritis Index (WOMAC)

The WOMAC [ICC = 0.96, 95% confidence interval (CI) 0.94–0.98] evaluates the pain, stiffness, and function of patients with knee osteoarthritis who have undergone TKA [30]. This tool has a total of 24 items, consisting of 5 pain items, 2 stiffness items, and 17 functional items. It is a five-point scale from 0 (lowest) to 4 (highest) for each item, with a lower score indicating fewer symptoms and less physical disability [31].

#### 2.7.2. Knee Flexion ROM

The self-passive knee flexion ROM was measured using a digital goniometer (Biometrics, Baton Rouge, LA, USA, 2008), which showed good intrarater ICC of 0.997–0.998 and interrater reliability ICC of 0.994 for knee joint ROM [32]. To measure the knee ROM, the axis of the digital goniometer was attached to the lateral joint space of the knee; the fixed arm was placed in the middle of the femur, between the greater trochanter and the lateral joint space of the knee; and the moving arm was lined up with the lateral malleolus of the fibula [33]. After the measurement, the angle of goniometer was reset to zero and the trial was repeated three times.

#### 2.7.3. Timed Up-and-Go (TUG) Test

The TUG test (ICC = 0.54–0.97) is used to assess dynamic balance ability and to predict falls in the elderly population [34]. In this study, the participants stood up from an armless chair upon the assessor’s signal, walked to the 3-m point, and returned to sit on the same chair. The test was performed three times, and the average value of the TUG time was obtained.

#### 2.7.4. Pressure Pain Threshold

The pressure pain threshold (kg/cm^2^) was measured using a digital pressure algometer (Pain Test™ FPX 25 Algometer; Wagner Instrument, Greenwich, CT, USA, 2015) at the vastus medialis; a position four fingers above the medial epicondyle. The measurement was performed while avoiding the surgical site (Cronbach’s alpha = 0.94–0.98) [35]. The average value of three trials was used in the analysis.

### 2.8. Data Analysis

The Kolmogorov–Smirnov test was used to ensure the normal distribution of the data on clinical and general characteristics. Repeated-measure analysis of variance (ANOVA; two-way, mixed-model) was performed to compare differences among three time points within groups and between groups, and a post hoc test was used with the Bonferroni method. Statistical analysis was performed using the Statistical Package for the Social Sciences (version 19; IBM, Chicago, IL, USA), and statistical significance was set at *p* < 0.05. Data are presented as means and standard deviations.

## 3. Results

A total of 237 participants were recruited. Of them, 60 patients who met the inclusion criteria and provided informed consent were randomized into the following groups: experimental 1 (preoperative telerehabilitation) group, experimental 2 (patient education) group, and control (usual care) group. The flow of patients throughout the study is shown in Figure 1. All participants were women aged >65 years with Kellgren–Lawrence grade 3–4 osteoarthritis that underwent TKA. The patient demographics are shown in Table 1.

### 3.1. Primary Outcome

#### Quadriceps Strength

**Figure 2:**
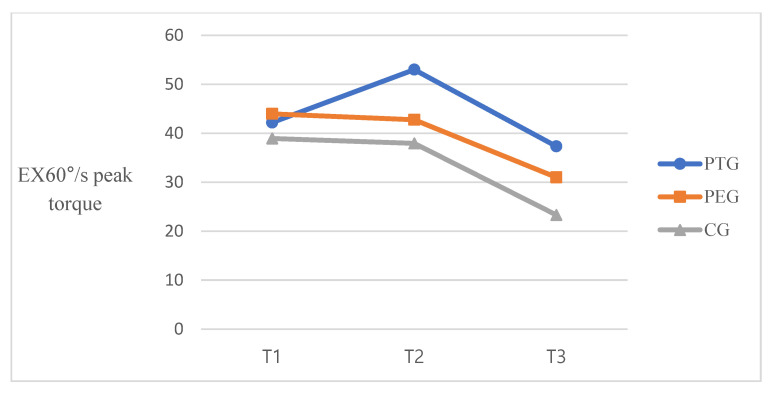
Isokinetic quadriceps strength at three-time points. T1, baseline; T2, post-intervention; T3, 6 weeks post-TKA.

Significant differences were observed across the three time points in 60°/s extension peak torque [F(2, 100) = 26.266, *p* < 0.001, *η^2^_p_* = 0.344]. Significant differences between groups were also observed [F(2, 50) = 8.625, *p* < 0.001, *η^2^_p_* = 0.256], as well as a significant time-by-group interaction [F(4, 100) = 2.499, *p* = 0.047, *η^2^_p_* = 0.91]. For 180°/s extension peak torque, there was a significant difference in time effect [F(2, 100) = 31.373, *p* < 0.001, *η^2^_p_* = 0.386], as well as significant differences between groups [F(2, 50) = 6.039, *p* = 0.004, *η^2^_p_* = 0.195] and a significant time-by-group intervention interaction [F(4, 100) = 3.583, *p* = 0.009, *η^2^_p_* = 0.125] (Table 2). Figure 2 is represented the trend of quadriceps strength at three-time points.

### 3.2. Secondary Outcomes

#### 3.2.1. Knee Flexion ROM, Pain and Dynamic Balance

**Figure 3:**
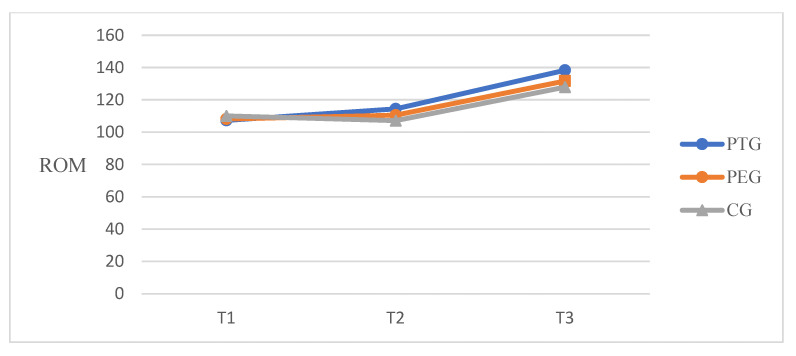
Knee flexion ROM at three-time points. T1, baseline; T2, post-intervention; T3, 6 weeks post-TKA.

A significant time effect on self-passive knee flexion ROM [F(2, 100) = 206.71, *p* < 0.001, *η^2^_p_* = 0.805] and significant differences between groups [F(2, 50) = 3.907, *p* = 0.027, *η^2^_p_* = 0.135] were found. A significant time-by-group interaction was also observed [F(4, 100) = 4.689, *p* = 0.002, *η^2^_p_* = 0.158]. Figure 3 is showed the trend of knee flexion ROM at three-time points. There was a significant time effect on the TUG test time [F(2, 100) = 45.559, *p* < 0.001, *η^2^_p_* = 0.477], as well as significant differences between groups [F(2, 50) = 3.584, *p* = 0.035, *η^2^_p_* = 0.125] and a significant time-by-group interaction [F(4, 100) = 7.252, *p* < 0.001, *η^2^_p_* = 0.225]. For pressure pain threshold at the three time points, there was a significant time-by-group intervention interaction [F(2, 100) = 7.78, *p* < 0.001, *η^2^_p_* = 0.135], whereas there were no significant differences between groups [F(2, 50) = 2.261, *p* = 0.115, *η^2^_p_* = 0.083] and no significant time-by-group intervention interaction [F(4, 100) = 0.900, *p* = 0.467, *η^2^_p_* = 0.035] (Table 3).

#### 3.2.2. Self-Reported Questionnaires

**Figure 4:**
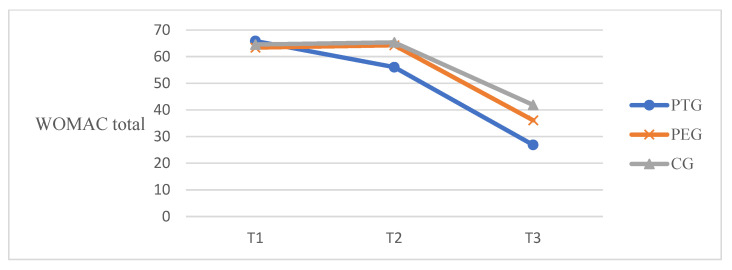
WOMAC total score at three time points. T1, baseline; T2, post-intervention; T3, 6 weeks post-TKA.

According to the self-reported WOMAC questionnaires, there was a significant time effect on pain score [F(2, 100) = 293.96, *p* < 0.001, *η^2^_p_* = 0.855], significant differences between groups [F(2, 50) = 7.412, *p* = 0.002, *η^2^_p_* = 0.229], and a significant time-by-group interaction [F(4, 100) = 9.113, *p* < 0.001, *η^2^_p_* = 0.267]. For the stiffness score, the time effect was significantly different [F(2, 100) = 124.36, *p* < 0.001, *η^2^_p_* = 0.713]; however, there was no significant difference between groups [F(2, 50) = 1.809, *p* = 0.174, *η^2^_p_* = 0.067]. The time-by-group interaction showed a significant difference [F(4, 100) = 5.33, *p* < 0.001, *η^2^_p_* = 0.176]. For functional score, there was a significant difference in the time effect [F(2, 100) = 273.75, *p* < 0.001, *η^2^_p_* = 0.847] and a significant difference between groups [F(2, 50) = 8.346, *p* < 0.001, *η^2^_p_* = 0.250]. The time-by-group interaction showed a significant difference [F(4, 100) = 6.579, *p* < 0.001, *η^2^_p_* = 0.208]). For total WOMAC total score, there was significant difference the time effect, F(2, 100) = 420.210, *p* < 0.001, *η^2^_p_* = 0.894, and significant differences between groups, F(2, 50) = 12.582, *p* < 0.001, *η^2^_p_* = 0.335. The time-by group interaction observed a significant difference F(4, 100) = 10.410, *p* < 0.001, *η^2^_p_* = 0.294 (Table 4). Figure 4 is represented the trend of WOMAC total score at three-time points.

## 4. Discussion

This study aimed to investigate the effect of a preoperative telerehabilitation program on muscle strength, ROM, and functional outcomes in patients undergoing TKA. The findings of this study indicated that the preoperative telerehabilitation program had a positive effect on the outcomes after TKA. It improved the patient-reported functional outcomes, muscle strength, and ROM of patients with end-stage osteoarthritis who underwent TKA. The patients reported better pain scores at 6 weeks after TKA. In contrast, no close relationship was found between patient-reported pain and pressure pain threshold at the three time points.

A. Isokinetic Muscle Strength

Reduced quadriceps strength is commonly observed in patients immediately after TKA, and it takes from 6 months to 1 year to regain the preoperative values [36]. Weakness of the quadriceps affects neuromuscular activation and functional ability, leading to an increased risk of falls in patients undergoing TKA [37]. High-intensive preoperative training that included resistance training exercise and strengthening exercise for 3 days per week for 8 weeks improved isometric knee extension value after training and 3 months after TKA compared with the control group [26]. Similarly, Swank et al. reported on the prehabilitation program that included resistance training with elastic bands, flexibility, and step training 3 times per week for 4–8 weeks significantly improved knee extension peak torque in the surgical leg after the intervention [38]. In this study, the intervention protocol included training of knee extensors and resistance exercise with Thera-Band. It may impact increasing knee extension peak torque after intervention and 6 weeks after TKA. The extension strength of the PT group and PE group increased after intervention and had decreased less after 6 weeks TKA compared to the control group. Compared to the mean value of 60°/s extension peak torque between groups, the PT group and PE showed a decrease of 11.5% and 11.37%, respectively, whereas the control group showed 40.13% at baseline to post-TKA. In 180°/s extension peak torque, the TP and PE groups showed a decrease of 6.17% and 22.98%, respectively, whereas the control group showed 31.9% at baseline to post-TKA. Although the PT group spent more time exercising than the PE group, this result was probably due to the protocol of the prehabilitation program being the same and the motivation and education successfully provided in both groups by physical therapists. The less decreased extension strength was a significant predictor for recovery of postoperative muscle strength and the possibility of easy regain of preoperative values. We did not consider minimal clinical important difference (MCID) in muscle strength because there was no evidence in our candidates for TKA [39].

B. Functional outcomes

A patient with end-stage knee osteoarthritis experiences severe pain when in a weight bearing position and experiences physical function difficulties when performing everyday activities along with weakness of leg strength [40]. Pain and limitation of physical function in osteoarthritis patients eventually decrease the patient’s quality of life, which is a major reason for patients to consider TKA. After successful TKA, a decrease in pain and improvement of physical function promotes functional activities and ADL such as walking, climbing, sit-to-stand movement, self-care, and housekeeping. The preoperative knee ROM, pain, and functional ability predict postoperative outcomes among patients undergoing TKA [41]. The present study conducted tele-prehabilitation that consists of mobility, flexibility, strengthening exercise (using elastic band) and balance exercise for 3 weeks before TKA and found improvements in all outcomes such as knee ROM, WOMAC points, and TUG time. The knee flexion ROM was measured self-passively using a digital goniometer. This increased from 107° to 114° at pre-post intervention and 138° at 6 weeks after TKA in the PT group, from 108° to 110° at pre-post intervention and 131° at 6 weeks after TKA in the PE group. The control group increased from 110° to 107° at pre-post intervention and was measured as 127° at 6 weeks after TKA. The PT group and PE group had been trained in knee ROM exercise and quadriceps stretching and these may be influenced by the increasing knee ROM in comparison to the control group. Regarding the WOMAC score, a significant difference was reported in the time-by-group interaction of all subscales and total score. The minimal clinical important improvement (MCII) for WOMAC recommended a 12% improvement from baseline for OA [42]. The baseline value of the PT group was 65.89 and improved 9.83 points at pre-post intervention over more than 7.9 (12%). Although the self-reported WOMAC pain score differed significantly, the PPT showed no effect. In TUG time, the minimal detectable change (MDC) is recommended as 1.14 s for OA [43] and 2.27 s for TKA [44]. The TUG time in the PT group showed a meaningful decrease of 3.23 s compared to the PE group of 1.12 s and the control group of −0.33 s at pre-post intervention. The TUG time decreased by 4.03 s in the PT group, 2.83 s in the PE and 1.36 s in the control group at baseline to 6 weeks post-TKA. The mini squat may promote sit-to-stand movement and help practice timed up-and-go tests. The fall incidents reported one case (5%) in the control group. Therefore, the protocol of this preoperative telerehabilitation program may have positive effects on the ability of patients’ balance in the early stages after TKA. Similar to the results of this study, Vasta et al. reported in their systematic review that preoperative physical activity or prehabilitation (including muscle strengthening with or without elastic resistance, proprioceptive exercise, progressive resistance training, and home-based exercise program) at 6 and 3 weeks before TKA in elderly patients improved the pain score, ROM, and functional index compared with the control group [13]. The study by Chughtai et al. (*n* = 114) demonstrated that patients in the prehabilitation with telerehabilitation group had shorter LOS than the control group [24]. LOS was not measured in the current study because all countries have different healthcare systems, and patients after bilateral TKA typically stay in the hospital for 3 weeks. In a meta-analysis of randomized controlled trials, Chen et al. reported that the prehabilitation group had better outcomes in knee ROM, LOS, and sit-to-stand test than the control group [18]. Despite the benefits of preoperative intervention, some studies have reported no significant difference in quadriceps strength and physical function. In the study by Beaupre et al., 66 patients underwent 6 weeks of preoperative exercise and education before TKA. The study found no difference in knee ROM, WOMAC, and strength between the two groups [15]. Devasenapathy et al. also found that preoperative muscle strength and function had a poor agreement with the outcomes after TKA, and the effect of preoperative intervention lasted for only 6 months to 1 year. Intervention protocols, such as intervention intensity, frequency, duration, and follow-up duration, may contribute to the outcomes. The duration of the intervention varied from 2 to 12 weeks, and the frequency was mainly 2–3 times a week [12]. In this study, we provided 30 sessions for 3 weeks as intensive training for elderly patients. The approaching date of surgery provided sufficient motivation to induce the patient’s commitment to treatment, however, two participants discontinued their participation due to the high intensity. We did not verify the long-term effects of preoperative telerehabilitation but observed improvements in physical function and restoration of muscle strength in the early phase after TKA.

This study had several limitations. First, the sample size was small and only female participants were included. Although the results of the present study showed improvements in muscle strength and functional outcomes, a large sample size is required to provide definitive evidence on the effect of preoperative telerehabilitation. Moreover, our sample consisting of female patients does not represent the typical cases. In addition, this study provides only data on the short-term outcomes (at 6 weeks after TKA) of preoperative telerehabilitation, and we could not control for the use of pain medications in the early phase after TKA. Therefore, long-term follow-up is required to prove the independent effects on pain, excluding the bias of this program. This study attempted to maximize the treatment effect by applying real-time telerehabilitation, and there was a limitation in the composition of the protocol as a non-face-to-face intervention that prioritized the safety of elderly patients with end-stage osteoarthritis. Further studies with a large sample size and including typical participants are needed. Furthermore, long-term follow-up data are required before the broad application of this preoperative telerehabilitation program.

## 5. Conclusions

The present study demonstrates the early benefits of a preoperative telerehabilitation program in elderly female patients with end-stage osteoarthritis. This preoperative telerehabilitation program improved muscle strength, ROM, and functional outcomes in patients before TKA, which contributed to better functional recovery after TKA.

## Figures and Tables

**Table 1:** Demographics of the participants.

Characteristics	PTG (*n* = 18)	PEG (*n* = 17)	Control (*n* = 18)	*X^2^* F(*p*)
Age (years)	71.1 ± 3.30	70.05 ± 2.41	70.38 ± 2.59	0.650 (0.527)
Height (cm)	152 ± 6.35	151.58 ± 6.03	151.70 ± 5.58	0.109 (0.897)
Weight (kg)	61.75 ± 7.97	60.47 ± 9.29	61.95 ± 8.62	0.146 (0.865)
BMI (kg/m²)	26.45 ± 2.50	26.25 ± 3.26	26.51 ± 2.86	0.176 (0.839)
BMD (T-score)	−2.27 ± 1.03	−2.58 ± 0.91	−2.40 ± 0.93	0.474 (0.626)
Deviation of the knee joint axis (°)	15.70 ± 6.29	11.17 ± 5.48	14.44 ± 1.49	2.636 (0.082)

PTG, preoperative telerehabilitation group; PEG, preoperative patient education group; BMI, body mass index; BMD, bone mineral density. Values are expressed as mean ± standard deviation. Significant: *p* < 0.05.

**Table 2:** Quadriceps strength measured over time (*n* = 53).

	PTG (*n* = 18)	PEG (*n* = 17)	Control (*n* = 18)	Time F(*p*)	Group F(*p*)	Group × Time Interaction F(*p*)
EX60°/s peak torque (N-m)				26.26 (0.001)	8.63 (0.001)	2.49 (0.047)
Baseline	42.19 ± 12.41	34.98 ± 14.16	38.95 ± 17.13			
Postintervention	53.071 ± 9.71	42.77 ± 9.72	37.94 ± 15.61			
6 weeks post-TKA	37.34 ± 5.12	31.01 ± 6.88	23.32 ± 5.15			
Ex180°/s peak torque (N-m)				31.37 (0.001)	6.04 (0.004)	3.58 (0.009)
Baseline	25.69 ± 10.49	24.59 ± 9.29	25.93 ± 10.57			
Postintervention	37.28 ± 5.79	29.31 ± 7.94	25.47 ± 8.75			
6 weeks post-TKA	24.13 ± 4.09	18.94 ± 4.31	17.64 ± 5.25			

PTG, preoperative telerehabilitation group; PEG, preoperative patient education group; EX, extension. Values are expressed as mean ± standard deviation. Significant: *p* < 0.05 F: two-way repeated-measure analysis of variance.

**Table 3:** ROM, PPT and TUG time measured over time (*n* = 53).

	PTG (*n* = 18)	PEG (*n* = 17)	Control (*n* = 18)	Time F(*p*)	Group F(*p*)	Group × Time Interaction F(*p*)
Knee flexion ROM (°)				206.70 (0.001)	3.91 (0.027)	4.69 (0.002)
Baseline	107.32 ± 7.09	108.16 ± 9.92	110.05 ± 10.40			
Postintervention	114.41 ± 6.26	110.52 ± 8.84	107.18 ± 10.23			
6 weeks post-TKA	138.32 ± 1.79	131.70 ± 3.18	127.91 ± 6.73			
TUG time (s)				45.56 (0.001)	3.58 (0.035)	7.25 (0.001)
Baseline	13.84 ± 2.48	13.27 ± 2.71	13.01 ± 1.84			
Postintervention	10.61 ± 1.26	12.16 ± 1.60	13.34 ± 2.13			
6 weeks post-TKA	9.81 ± 1.06	10.45 ± 0.67	11.65 ± 1.85			
PPT (kg/cm^2^)				7.78 (0.001)	2.26 (0.115)	0.9 (0.467)
Baseline	2.47 ± 0.67	2.89 ± 0.73	2.65 ± 0.54			
Postintervention	2.58 ± 0.76	2.64 ± 0.78	2.81 ± 0.84			
6 weeks post-TKA	3.11 ± 0.92	3.57 ± 0.96	3.01 ± 1.09			

PTG, preoperative telerehabilitation group; PEG, preoperative patient education group; PROM, passive range of motion; PPT, pressure pain threshold. Values are expressed as mean ± standard deviation. Significant: *p* < 0.05. F: two-way repeated-measure analysis of variance.

**Table 4:** WOMAC score measured over time (*n* = 53).

	PTG (*n* = 18)	PEG (*n* = 17)	Control (*n* = 18)	Time F(*p*)	Group F(*p*)	Group × Time Interaction F(*p*)
WOMAC total				420.21 (0.001)	12.58 (0.001)	10.41 (0.001)
Baseline	65.87 ± 7.34	63.41 ± 7.16	64.56 ± 8.75			
Postintervention	56.06 ± 5.25	64.29 ± 5.95	65.39 ± 6.38			
6 weeks post-TKA	26.89 ± 4.30	36.12 ± 5.04	41.89 ± 8.24			
WOMAC pain				293.96 (0.001)	7.41 (0.002)	9.113 (0.001)
Baseline	12.66 ± 1.72	11.58 ± 1.58	12.11 ± 1.7			
Postintervention	11.00 ± 1.14	11.53 ± 2.57	12.67 ± 1.6			
6 weeks post-TKA	3.50 ± 1.33	6.00 ± 1.00	7.05 ± 2.55			
WOMAC stiffness				124.37 (0.001)	1.80 (0.174)	5.33 (0.001)
Baseline	4.89 ± 1.07	4.77 ± 1.39	4.39 ± 0.92			
Postintervention	4.38 ± 1.03	4.82 ± 1.23	4.56 ± 0.85			
6 weeks post-TKA	1.56 ± 0.51	2.23 ± 0.44	2.95 ± 0.83			
WOMAC function				275.75 (0.001)	8.34 (0.001)	6.57 (0.001)
Baseline	48.33 ± 7.02	47.05 ± 6.14	48.05 ± 7.08			
Postintervention	40.67 ± 4.81	47.94 ± 4.52	48.16 ± 6.34			
6 weeks post-TKA	21.83 ± 4.31	27.88 ± 4.47	31.89 ± 6.16			

PTG, preoperative telerehabilitation group; PEG, preoperative patient education group; WOMAC: Western Ontario and McMaster Universities Osteoarthritis Index; Values are expressed as mean ± standard deviation. Significant: *p* < 0.05. F: two-way repeated-measure analysis of variance.

## Data Availability

Not applicable.

## Appendix A. Protocol of the Preoperative Telerehabilitation Program

**Exercise**	**Explanation**	**Figure**
Straight leg raises	In the supine position, pull the ankle toward the head and raise one leg 45° upward. Relax after holding for 5 s. Repeat with the other leg. 10 repetitions/1 set	
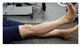
Bridges	In the supine position, keep the feet flat on the floor with the knees bent. Raise the buttocks toward to ceiling. Relax after holding for 5 s. 10 repetitions/1 set	
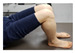
Leg slides (supine position)	Slide a leg out to the side, keeping the kneecap pointed up toward the ceiling. Slide the leg back to the starting position. 10 repetitions/2 sets	
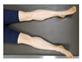
Ankle pump (supine position)	Attach an elastic band to the ankle and hold it with both hands. While extending the leg, push the foot opposite direction. 10 repetitions/2 sets	
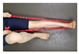
Knee presses	In the long sitting position, place a rolled towel under the ankle and press the knee down. Relax after holding for 5 s. Repeat with the other leg. 10 repetitions/2 sets	
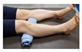
Passive ROM	In the sitting position, hold one knee with both hands and passively flex the knee such that the heel touches the thigh. 10 repetitions/1 set	
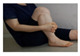
Mini squats	Stand with the feet shoulder-width apart and hold the back of a chair. Slowly bend the knees about 5 cm. After holding for 5 s, slowly straighten the knees. 10 repetitions/1 set	
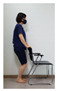
Quadriceps stretching	Stand behind the back of a chair. Take one foot back and fix the other knee slightly on the floor. Move the body forward while feeling the stretching of the back thigh muscles. Hold for 5 s, relax, and repeat with the other leg. 10 repetitions/1 set	
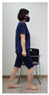
Hamstring stretching	Sit on the edge of the chair. Straighten one leg with the toes facing upward and the heel touching the floor. Slowly lean forward while keeping the back straight. After holding for 5 s, repeat with the other leg. 10 repetitions/1 set	
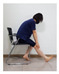
Leg slides (standing)	Hold the back of a chair and stand upright. While keeping the toes from falling to the floor, slide one foot backward until the hip muscles are tense. After holding for 5 s, return to the starting position. Repeat with the other leg. 10 repetitions/1 set	
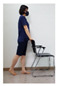
Quadriceps strength I	While sitting on a chair, wrap elastic bands on both ankles. Fix one foot to the floor to prevent dragging. Extend the other knee forward and hold the position for 5 s. After returning to the starting position, repeat with the other leg. 10 repetitions/2 sets	
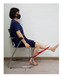
Quadriceps strength II	While sitting on a chair, wrap elastic bands on both ankles. Fix one foot to the floor to prevent dragging. Bend the other leg toward the chair and hold the position for 5 s. After returning to the starting position, repeat with the other leg. 10 repetitions/2 sets	
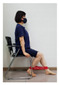
Abductor strength	While sitting on a chair, wrap elastic bands on both knees while kept together. Spread both legs at the same time. After holding for 5 s, return to the starting position. 10 repetitions/2 sets	
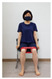
Tandem gait	Hold the wall railing with one hand and walk with the heel of one foot in line with the forefoot of the other. 10 repetitions/1 set	
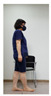
Trunk rotation	Rotate the trunk to the left and right while in a sitting position. 10 repetitions/1 set	
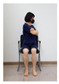
Total exercise time: 30 min (warm up, main exercise, cool down). Rest time: 20 s.		
